# Antimicrobial and acaricide sanitizer tablets produced by wet granulation of spray-dried soap and clove oil-loaded microemulsion

**DOI:** 10.1371/journal.pone.0313517

**Published:** 2024-11-11

**Authors:** Idejan P. Gross, Ana Luiza Lima, Evalina C. Sousa, Maiane S. Souza, Marcilio Cunha-Filho, Izabel Cristina Rodrigues da Silva, Daniela Castilho Orsi, Livia L. Sá-Barreto

**Affiliations:** 1 Laboratory of Food, Drugs and Cosmetics (LTMAC), School of Health Sciences, University of Brasilia, Brasília, DF, Brazil; 2 Faculty of Ceilandia, University of Brasilia (UnB), Brasília, DF, Brazil; Universidad Autonoma de Chihuahua, MEXICO

## Abstract

A novel sanitizer tablet containing clove essential oil (CO) microemulsion was developed. A preformulation study using nuclear magnetic resonance and thermal analyses showed component compatibility. The main components of the samples remained intact despite a color change, probably due to a strong acid-base interaction between eugenol and diethanolamine. The CO microemulsion showed acaricidal and larvicidal activities superior to the commercial product, with product efficacy of 99.9% and larvae mortality of 94%. Optimal spray-drying conditions were achieved with inlet and outlet temperatures of 50°C and 40°C, respectively, an aspiration rate of 1 m^3^ min⁻^1^, and a 0.25 L h⁻^1^ injection flow. The feed suspension comprised 50% (v/v) liquid soap, 37.5% (v/v) water, 12.5% (v/v) ethanol, and 5.0% (w/v) silica. This formulation and processing parameters allowed for successful free-flow powder formation, providing a suitable matrix for incorporating the CO microemulsion via wet granulation without heating. Finally, sanitizer tablets produced from such granules resulted in a uniform product with low weight variation (coefficient of variation of 0.15%), eugenol content of 95.5% ± 3.3, and friability of 0.58%. Furthermore, the tablets showed rapid aqueous dispersion, forming a colloidal system with particle sizes of 221 nm and a zeta potential of -17.2 mV. Antimicrobial activity tests demonstrated the effectiveness of the sanitizer tablet against bacteria and fungi, exhibiting comparable antimicrobial potency to isolated CO. Hence, the sanitizer tablet developed represents a promising candidate as a practical and efficient solution for pest control, offering strong antimicrobial and acaricidal activity.

## 1. Introduction

Essential oils have attracted attention for their antimicrobial properties [1,2]. Their effectiveness against a broad range of pathogens has sparked interest in incorporating them into sanitizer products [3]. In addition to their antimicrobial applications, essential oils have been studied for their insecticidal, repellency, and acaricidal properties [4–9]. Accordingly, several applications have been evaluated, from those involving local asepsis of surfaces [10,11] to applications such as insect repellency [12,13]. Such versatility makes essential oils potential candidates in preventing a wide variety of diseases, whether through its direct action against microorganisms or even in combating vectors.

*Syzygium aromaticum*, popularly known as cloves, is a rich source of clove essential oil (CO) containing phenolic compounds such as eugenol, which demonstrates diverse biological activities such as antibacterial, antifungal, insecticidal, and acaricidal [6,7,10,14–16]. Recognized by the FDA as generally safe (GRAS), CO has been used in the perfumery, cosmetics, pharmaceuticals, and the food industry [17–19].

The volatile nature of essential oil active components poses a significant obstacle to their industrial exploitation [20]. Moreover, many botanical compounds are highly prone to degradation through various environmental factors. For example, pyrethrins are susceptible to photodegradation, azadirachtins can undergo abiotic oxidation, and terpenoids are often lost through volatilization [4]. This vulnerability needs to be considered when choosing manufacturing processes that include natural products. In this sense, techniques such as the emulsification or nanoencapsulation of essential oils have been recently studied to protect and enable the use of these components [21–24].

The most common emulsification process involves the dispersion of the oil phase within an aqueous medium facilitated by surfactants or emulsifiers. Renex95® (RX95) is a non-ionic surfactant that acts to lower the interfacial tension between oil and water phases, promoting the formation of fine oil droplets dispersed in the continuous aqueous phase and enabling the creation of stable emulsions with reduced droplet coalescence [25,26]. Amida60® (AM60) is a coconut fatty acid diethanolamide compound that complements this process by acting as a co-emulsifier, viscosity modifier, and stabilizer [27]. Together, such compounds could have complementary actions playing a crucial role in achieving the desired texture, stability, and functionality of oil-in-water microemulsions.

The industrial viability of stabilized essential oil-based microemulsions still involves their incorporation into solid matrices that can protect the active ingredients from degradation caused by environmental factors such as light, air, and temperature fluctuations [28,29], while at the same time mitigating the risk of volatilization [30,31]. Moreover, this approach must be based on industrial procedures that streamline storage, transportation, and handling, making them conducive for commercial distribution and final use.

Therefore, this work aimed to develop a sanitizer tablet by incorporating a CO-based microemulsion within a soap solid matrix that can disperse this natural product easily while preserving its stability and properties through viable industrial processes. For this, a preformulation study was performed to evaluate the compatibility between the CO and excipients by thermal analysis and nuclear magnetic resonance. Next, a CO microemulsion was obtained and incorporated into sanitizer tablets, which were characterized according to their physical-chemical properties, as well as acaricidal, larvicide, and antimicrobial activities.

## 2. Materials and methods

### 2.1. Materials

CO obtained by steam distillation of dried flower buds was purchased from Terra Flor (lot 21263, Alto Paraíso de Goiás, Brazil) containing as main components 80.2% eugenol, 13.2% beta-caryophyllene, 3.8% isoeugenyl acetate, and 1.3% alpha-humulene (gas chromatography). RX95 (nonylphenol ethoxylate, lot 16355) and AM60 (coconut fatty acid diethanolamide, lot 16183) were afforded by Casa da Quimica (Brasília, Brazil). Potassium hydroxide (KOH; lot 08498) was acquired from Quimica Moderna (Barueri, Brazil). Eugenol standard (lot BCCH3111) was purchased from Merck (Darmstadt, Germany). Aerosil® (silicon dioxide, lot 158070614) was afforded by Henrifarma (São Paulo, Brazil), deuterated methanol (CD3OD) 0.03% tetramethylsilane (lot MBBD4736) was purchased from Sigma Aldrich (Missouri, USA) and Triatox® (lot 03620) was obtained from Cobasi (Brasília, Brazil). All solvents were chromatographic grade and were purchased from Merck (Darmstadt, Germany). The water used in the experiments was ultrapure water grade Milli-Q® (Darmstadt, Germany).

### 2.2. Preformulation studies

#### 2.2.1. Sample preparation

The compatibility of CO with the formulation materials was performed using binary physical mixtures of the oil, and each main component was prepared in an equimass proportion (1:1; w/w) using grade and pistil [32].

#### 2.2.2. Differential Scanning Calorimetry (DSC)

DSC measurements were performed using a DSC-60A plus calorimeter (Shimadzu; Kyoto, Japan) coupled with an EK90 cooler (Thermo Fisher Scientific; Waltham, MA, USA) using approximately 3 mg of the binary mixtures and the pure materials. Samples were placed in aluminum hermetic pans to prevent evaporation of volatile components. The equipment operated under a nitrogen atmosphere (50 mL min^-1^) at 10°C min^-1^ from -20 to 140°C.

#### 2.2.3. Thermogravimetric Analysis (TGA)

TGA was performed using a DTG-60H (Shimadzu, Kyoto, Japan) operating from 25 to 500°C at a heating rate of 10°C min^-1^, with approximately 5 mg of the binary mixtures and the pure materials placed in platinum pans. All analyses were conducted under a nitrogen atmosphere at a 50 mL min^-1^ flow.

#### 2.2.4. Fourier transform infrared spectroscopy (FTIR)

The infrared spectra were recorded on an infrared spectrometer (Bruker Vertex 70; Billerica, MA, USA) using the equipment ATR accessory. All samples were analyzed within the region of 4000 cm^− 1^ to 750 cm^−1^ and using a resolution of 2.0 cm^−1^, with 96 scans.

#### 2.2.5. Nuclear magnetic resonance (NMR) spectroscopy

NMR spectra were recorded on an AscendTM 600 MHz NMR spectrometer (Brucker; Billerica, MA, USA). The samples were dissolved in CD_3_OD with tetramethylsilane as an internal standard. The ^1^H and ^13^C NMR spectra were, respectively, obtained with 16 and 4096 scans in a spectral width of 20.1 and 238.9 ppm and 65536 raw data points.

A Distortionless Enhancement by Polarization Transfer (DEPT) experiment was also performed, with a flip angle of 135 (DEPT-135), aiming to distinguish the signals associated with CH, CH_2,_ and CH_3_ groups. Additionally, ^1^H–^13^C HSQC (Heteronuclear Single Quantum Coherence) two-dimensional spectrum was particularly useful for identifying which protons are directly bonded to which carbons, facilitating the assignment and, together with the above-mentioned techniques, elucidating the structure of the main components.

### 2.3. Microemulsion development

A blend of surfactants RX95 and AM60 in the ratio 3:1 w/w was initially prepared, combining the components under magnetic stirring. Subsequently, this mixture was incorporated in water, and then CO was introduced under magnetic stirring to obtain an oil-in-water microemulsion. Different amounts of CO were evaluated (5, 12.5, and 20%, w/v). Next, the samples underwent a 10min treatment in an ultrasonic bath [33].

### 2.4. Acaricidal activity

Approximately 450 engorged female ticks (*Rhipicephalus microplus*) were manually collected from naturally infested zebu cattle at the Dairy Zebu Breeds Technology Center of EMBRAPA (Brasília, Brazil). The cattle had not received any parasiticide treatment for at least 30 days before collection. The ticks were washed with clean running water, dried with absorbent paper, and selected based on vigor, motility, and weight. The acaricide test methodology followed the technique described by Drummond et al. (1973) [34].

The ticks were exposed to different treatments, including i) distilled water as a negative control, ii) commercial product (Triatox®) as a positive control, iii) pure CO, iv) surfactant-cosurfactant mixture (RX95:AM60 in a 3:1 ratio), and v) CO microemulsion. Thirty ticks exhibiting similar size and activity levels were selected for each treatment. They were immersed in the respective media for 5 min, then dried with absorbent paper and placed in Petri dishes with double-sided tape to secure them dorsally. Each group was labeled and kept in a climate-controlled chamber at 25°C and 70% relative humidity (RH) for 18 days (Model 420-CLDTS, Ethic; Vargem Grande Paulista, Brazil). All tests were performed in triplicate.

Acaricide efficacy was evaluated using the equations by Drummond et al. (1973) [34]:

$$RE=\frac{m_{eggs}\times \%Eclosion\times 20,000}{m_{fem}}$$

where RE is the reproductive efficacy, m_eggs_ is the egg mass, m_fem_ is the mass of the females, %hatching, and 20,000 represents the number of larvae per gram of eggs.

$$PE=\frac{\left({RE}_{control}-{RE}_{product}\right)}{{RE}_{control}}\times 100$$

where PE is the product efficacy, RE_control_ is the reproductive efficiency of the negative control, and RE_product_ is the reproductive efficiency of the tested product. Treatment efficacy was determined relative to the RE of the sterile distilled water control.

Treatments achieving a minimum RE of 95% were considered adequate following regulations for acaricide commercialization [35].

### 2.5. Larvae mortality

The larvae mortality was determined by placing females in Petri dishes in a dorsal position and keeping them in a climate chamber at approximately 25°C with 70% RH for 18 days. After this period, 50 mg of eggs were weighed and kept in a 2 mL Eppendorf tube until the larvae hatched.

Such a test was conducted for the same treatments cited in the evaluation of the acaricidal activity. For this purpose, 1.0 mL of each treatment solution was added to the respective groups, and the tubes were sealed and vigorously shaken using a vortex mixer for 5 min. After this time, the larvae were placed on filter paper using a brush, secured with a clip, and maintained at the same storage conditions.

The larvae immersion tests were based on the method described by Shaw (1966) [36], which was adapted for studies with oils and plant extracts [37]. Mortality was assessed 24h post-procedure, with live and dead larvae counted visually using a magnifying glass. The test was performed in triplicate for each treatment.

### 2.6. Soap synthesis

A saponification reaction was performed using recycling soy oil, blended with an aqueous solution containing KOH at a concentration of 1.16 g mL^-1^. A mixture of 50 mL of the strong base solution and 250 mL of oil was prepared using a mechanical stirrer (IKA; Staufen, Germany) for 30 min at room temperature.

The liquid soap underwent drying in an MSD 0.5 spray dryer (LabMaq; Ribeirão Preto, Brazil). Several methodologies were explored until the drying process was optimized, varying the inlet drying temperature, the injection flow, and drying support diluents, as well as their concentrations. The development of the drying conditions is shown in S1 Table. Ultimately, method 9 was chosen as the most effective powder soap production. The methodology consisted of an inlet temperature of 50°C, aspiration of 1 m3 min^-1^, injection flow of 0.25 L h^-1^, and airflow of 50 mL min^-1^. Furthermore, for the drying process, the soap was diluted, and the resulting sample comprised 50% soap, 37.5% water, 12.5% ethanol (v/v), and 5% Aerosil® (w/v) (S1 Table).

### 2.7. Sanitizer tablet development

A granule was initially prepared using the powder soap as a matrix and the CO microemulsion as a binding agent in a 1:1 w/v ratio to obtain the tablet. Then, the tablet was produced by compressing the granules using a Monopress LM-01 eccentric tablet press (Lemaq; Diadema, Brazil), equipped with round flat 10 mm punches. The tablets were designed to have a nominal mass of 250 mg, and the compaction force was adjusted to maintain tablet hardness within the specified range of 5–10 kgF.

### 2.8. Physicochemical characterizations

#### 2.8.1. Dynamic light scattering (DLS)

The microemulsion, soap, and tablet were dispersed in water to determine droplet size and polydispersity index (PDI). This characterization was conducted using a Zetasizer Nano ZS apparatus (Malvern; Malvern, United Kingdom). The light scattering of the droplets was assessed at a 90° angle at a temperature of 25°C in polystyrene cuvettes.

The chosen dilution for these analyses mirrored that of the final product, i.e., the dilution applied to use the sanitizer tablet. For that, 0.250 g of solid or semi-solid material (microemulsion, powdered soap, or tablet) was dispersed in 250 mL of water, resulting in a ratio of approximately 1:750 (w/v).

#### 2.8.2. Zeta potential

The zeta potential was determined through the electrophoretic mobility of the droplets. The analyses were conducted using the Zetasizer Nano ZS equipment (Malvern; Malvern, United Kingdom). The dilution described for DLS determination was also used for these analyses.

#### 2.8.3. Optical microscopy

The morphology of the powdered soap, the granules, and the sanitizer tablets was evaluated by optical microscopy using a SZ–SZT stereoscope (Laborana; São Paulo, Brazil) coupled with a video camera.

#### 2.8.4. Physical evaluation of the tablet

The mass uniformity of the sanitizer tablets was measured using the average of 10 tablets, which was determined by an analytical balance (Shimadzu; Kyoto, Japan). Friability was calculated as the percentage of mass loss from 10 tablets after 100 revolutions at 25 rpm using a friabilometer (Pharma Test; Hainburg, Germany). Furthermore, the hardness of 5 tablets was obtained using a TBH 100 durometer (Erweka; Langen, Germany).

#### 2.8.5. Eugenol determination

Quantification of eugenol in the sanitizer tablet was performed by a spectrophotometric method using a UV-VIS spectrophotometer UV-1800 (Shimadzu, Japan) operating from 700 to 190 nm. The combination of eugenol and AM60 allowed the identification at 493 nm. The method selectivity was tested against all excipients, showing no signal interference. The linearity correlation coefficient was 0.998, with a slope different from zero and residues randomly distributed without tendency.

### 2.9. Determination of antimicrobial activity

For the antimicrobial activity assays *Escherichia coli* (ATCC 25922), a gram-negative bacterium, *Staphylococcus aureus* (ATCC 25923), a gram-positive bacterium, and *Candida krusei* (ATCC 6258), a yeast, were used. The microorganism inoculum was prepared using direct suspension of microbial growth in Luria-Bertani broth (HiMedia; Kennett Square, PA, USA), with turbidity equivalent to 0.5 McFarland standard (108 CFU mL^-1^), adjusted between the optical density of 0.08 and 0.10 on a UV-Vis spectrophotometer UV-1800 (Shimadzu, Kyoto, Japan) in 625 nm. Then, the bacterial inoculum was diluted in this broth at a ratio of 1:150, resulting in a concentration of 106 CFU mL^-1^.

#### 2.9.1. Minimum inhibitory concentration (MIC), minimum bactericidal concentration (MBC), and minimum fungicidal concentration (MFC)

The MIC, MBC, and MFC were performed according to the Clinical and Laboratories Standards Institute method [38], with adaptations. Samples were diluted in broth in concentrations from 12.5 to 3.6 g L^-1^ (1.25 to 0.4%, w/v).

For MIC tests, 100 μL of different sample concentrations were distributed in a 96-well microtiter plate containing 100 μL of bacterial inoculum suspensions, which were incubated for 24 h at 37°C. The bacterial growth was assessed by adding 20 μL of a 0.01% w/v resazurin dye to each well. Such a component is a redox indicator employed to evaluate cell viability, which is initially blue and then turns pink when reduced to resorufin by oxidoreductases within viable cells [39]. Hence, the MIC was defined as the lowest concentration at which there was no change in color.

For MBC and MFC tests, 100 μL aliquots from each incubated sample were spread onto Mueller Hinton agar (HiMedia; Kennett Square, PA, USA) plates and incubated for 24 h at 37°C. The MBC and MFC were determined to be the lowest concentrations at which no visible bacterial or fungal growth was observed on the agar.

#### 2.9.2. Antimicrobial activity determined by the disk diffusion method

The disk diffusion method was performed following the Clinical and Laboratory Standards Institute method [40]. To perform the disk diffusion test, the microbial inoculum was seeded, with the aid of a sterile swab, on the surface of a Mueller Hinton (HiMedia; Kennett Square, PA, USA) agar plate until a uniform smear was obtained. After the inoculum was dried, different dilutions of the sanitizer tablet were applied (3.57, 4.17, 5.00, 6.25, 8.33, and 25.00 g L^-1^). The results were obtained after 24 h of incubation at 37°C by measuring the inhibition zones.

## 3. Results and discussion

### 3.1. Preformulation studies

The development of complex systems, including several processes and materials, such as in this study, presents an increased risk of incompatibilities that could ultimately compromise the stability of the final product. In this sense, detailed preformulation protocols are highly recommended to anticipate and prevent these occurrences [41].

**Fig 1:**
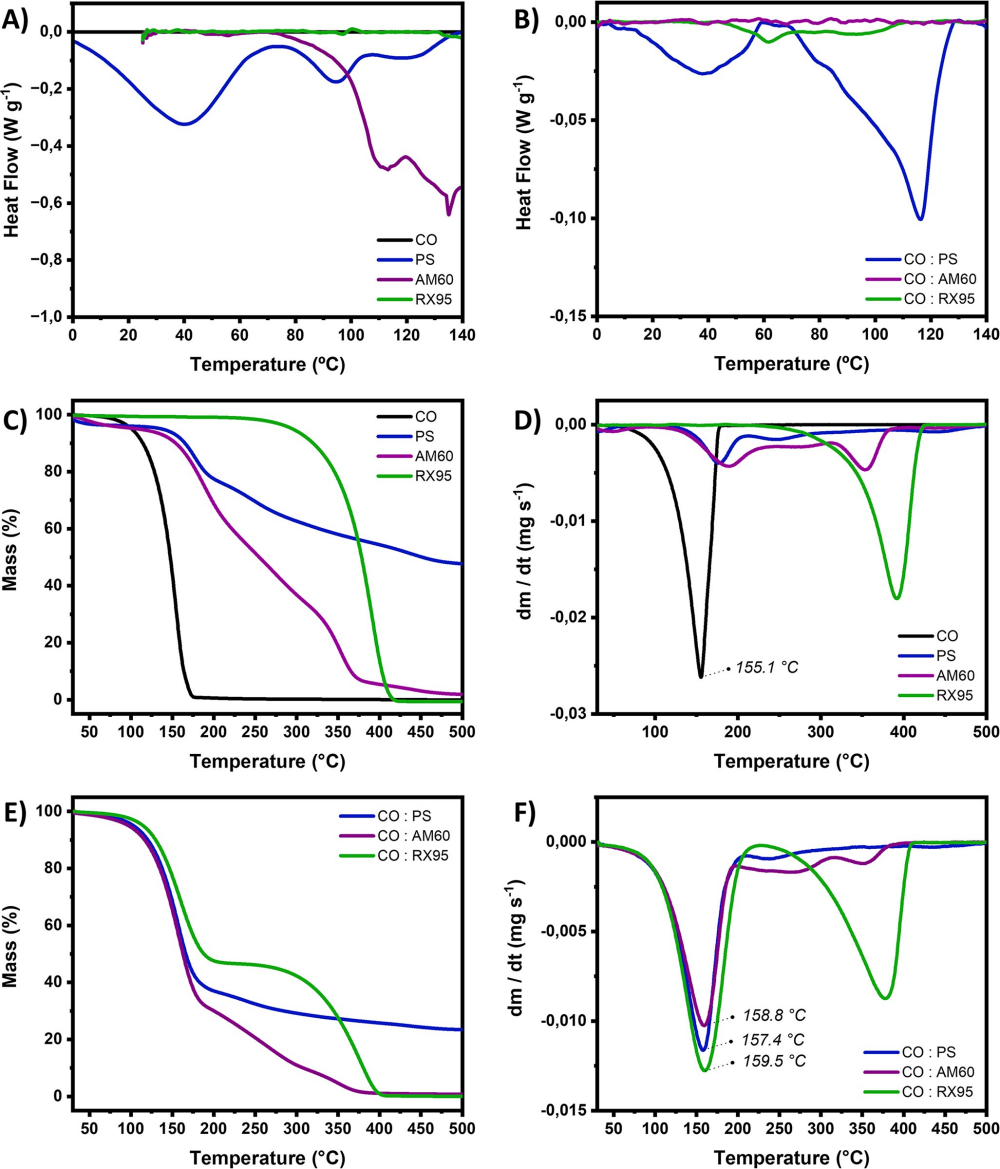
Thermal analyses performed for individual components and binary mixtures. DSC curves of **(A)** clove oil (CO), emulsion surfactants (RX95 and AM60), and the powder soap (PS) and **(B)** 1:1 (w/w) binary mixtures of CO with each formulation component. TGA (**C**) and DTG curves (**D**) of clove oil (CO), emulsion surfactants (RX95 and AM60), and powder soap (PS); and TGA (**E**) and DTG (**F**) curves of 1:1 (w/w) binary mixtures of CO with each formulation component.

Fig 1A illustrates the DSC curves of both the pure essential oil and the excipients alone (RX95, AM60, and powder soap). Within the temperature range of the analyses, no thermal events were observed for CO, corroborating its thermal stability. In fact, no peaks have been reported in the literature before 140°C for this material [42,43].

Three endothermic events were observed for powder soap (40°C, 95°C and 115°C), which can be attributed to the melting of some fatty acids and residual triglycerides from the saponification reaction (Fig 1A). Furthermore, an endothermic signal was observed for AM60, which can be associated to thermal degradation, in accordance with the mass loss seen in the TGA and DTG curves (Fig 1C and 1D). This thermal event is probably linked to the breakdown of the diethanolamine (DEA) component in AM60. These findings are consistent with Ávila et al. (2015), who reported that DEA decomposes thermally to produce monoethanolamine (MEA) and ethylene oxide [44].

**Fig 2:**
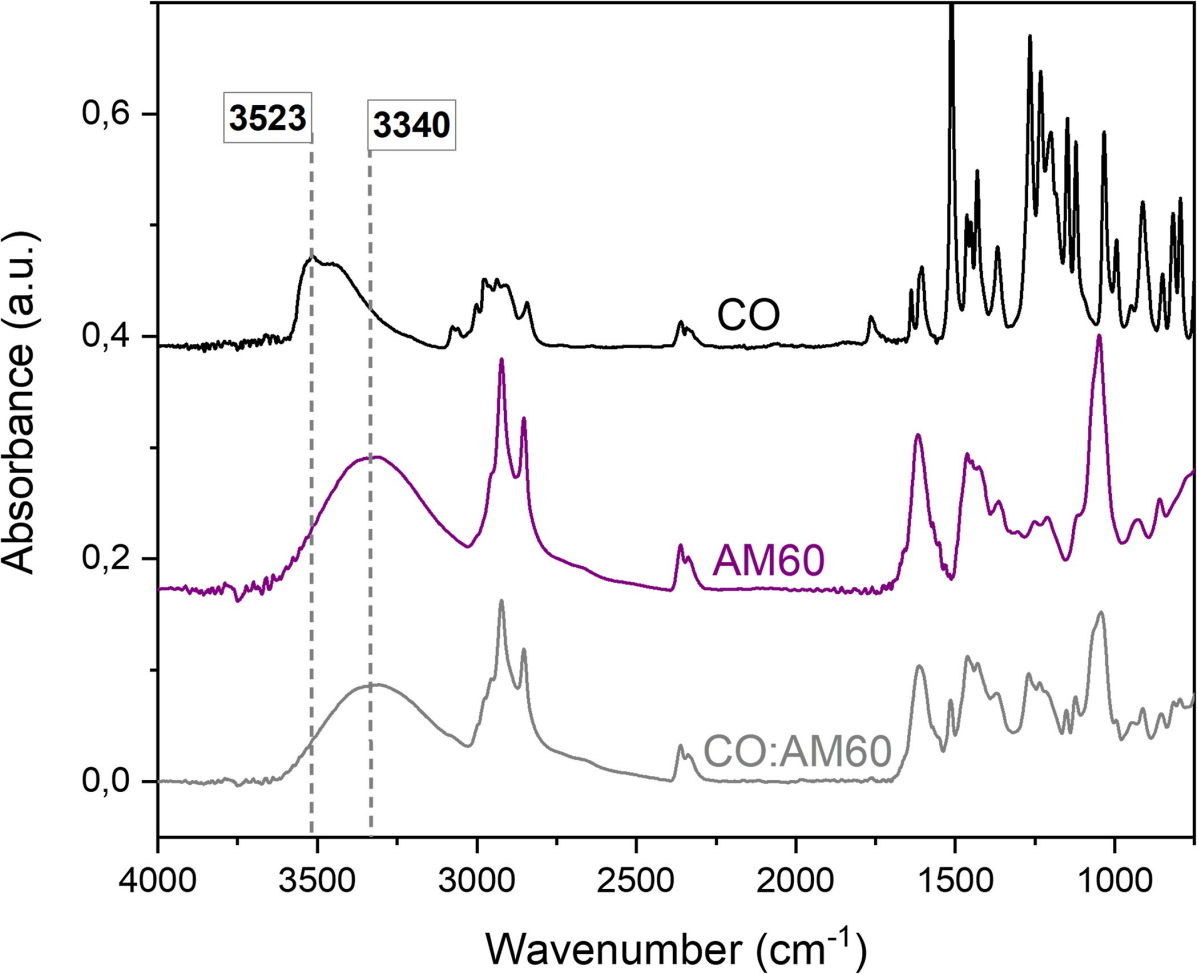
FTIR spectra evidencing the probable hydrogen bond formation between eugenol and diethanolamine. FTIR spectra of clove oil (CO), amide 60 (AM60), and 1:1 CO:AM60 binary mixtures, highlighting the OH stretching bands that show a red-shift, as a result of a hydrogen bond formation between eugenol in CO and diethanolamine in AM60.

No thermal events are identified for RX95 which appears as a homogeneous liquid (Fig 1A). The decomposition profile of the isolated components is shown in Fig 2A and 2B. A high residue was observed at the end of the heating cycle (500°C) for the powder soap, dried in a spray dryer. This is likely attributed to the use of Aerosil® in the liquid soap as a filler to optimize the drying process.

In binary mixtures (Fig 1E and 1F), the maximum mass loss rate temperature for the active ingredient (eugenol) remained unchanged at around 166°C. This observation suggests that the interaction of CO with other components in the formulation does not seem to impact the essential oil’s evaporation process significantly.

The presence of CO in the powder soap changed the melting behavior of the fatty acids and triglycerides (Fig 1B), suggesting that the components of the essential oil, mainly composed of eugenol, can act as plasticizers. Such an outcome has implications for the powder’s physical properties, potentially enhancing its agglomeration capacity during a granulation process and favoring tablet formation.

A subtle endothermic shift in the baseline for CO:RX95 was observed, suggesting a certain degree of interaction between the components, yet without significant repercussions (Fig 1B).

In turn, a notable change in the expected thermal profile was observed in CO:AM60 (Fig 1B). The endothermic event associated with the DEA thermal decomposition completely disappears in the CO:AM60 mixture, suggesting that some component of the clove oil may inhibit the DEA degradation.

The thermal degradation mechanism of DEA involves an intramolecular proton transfer from the hydroxyl group to the amine, resulting in the formation of the intermediate species HOEt–⁺NH₂–EtO⁻. This alkoxide group then nucleophilically attacks the carbon adjacent to the nitrogen, leading to cleavage of the C-N bond and subsequent formation of MEA and ethylene oxide [44].

It is possible, therefore, that the eugenol phenol group competes for the amine protonation, which inhibits the alkoxide formation and consequently prevents its intramolecular nucleophilic attack and the breakdown of the DEA molecule. Additionally, when CO (slightly yellowish) is combined with AM60 (light yellow), an instantaneous transformation into a distinctly orange liquid was observed (S1 Fig), probably associated with a eugenol–DEA acid-base complex formation.

In fact, several studies reported strong interactions between amine/ammonium and phenol/phenolate compounds [45–47]. Such interactions have been used to obtain deep eutectic solvents, generally based on a two or three-component eutectic mixture composed of hydrogen bond donors and acceptors at a certain stoichiometric ratio [48]. In this context, FTIR analyses were conducted to investigate the phenol OH stretching band (Fig 2).

The results show that OH stretching of the phenol was redshifted from 3523 cm^-1^ to 3340 cm^-1^, which evidences the strong hydrogen bonds between the compounds since not even shoulders were observed in the respective OH band for the CO:AM60. Similar results were already reported by Shen, et al. (2023) studying eugenol-ammonium salts interactions, where OH stretching was redshifted from 3542 cm^-1^ to 3440 cm^-1^ [48].

Additionally, CO:AM60 was chosen for a comprehensive NMR analysis, aiming to confirm that the chemical structure of eugenol is maintained and no covalent bonds are formed or broken, which could affect the performance of the active ingredient.

**Fig 3:**
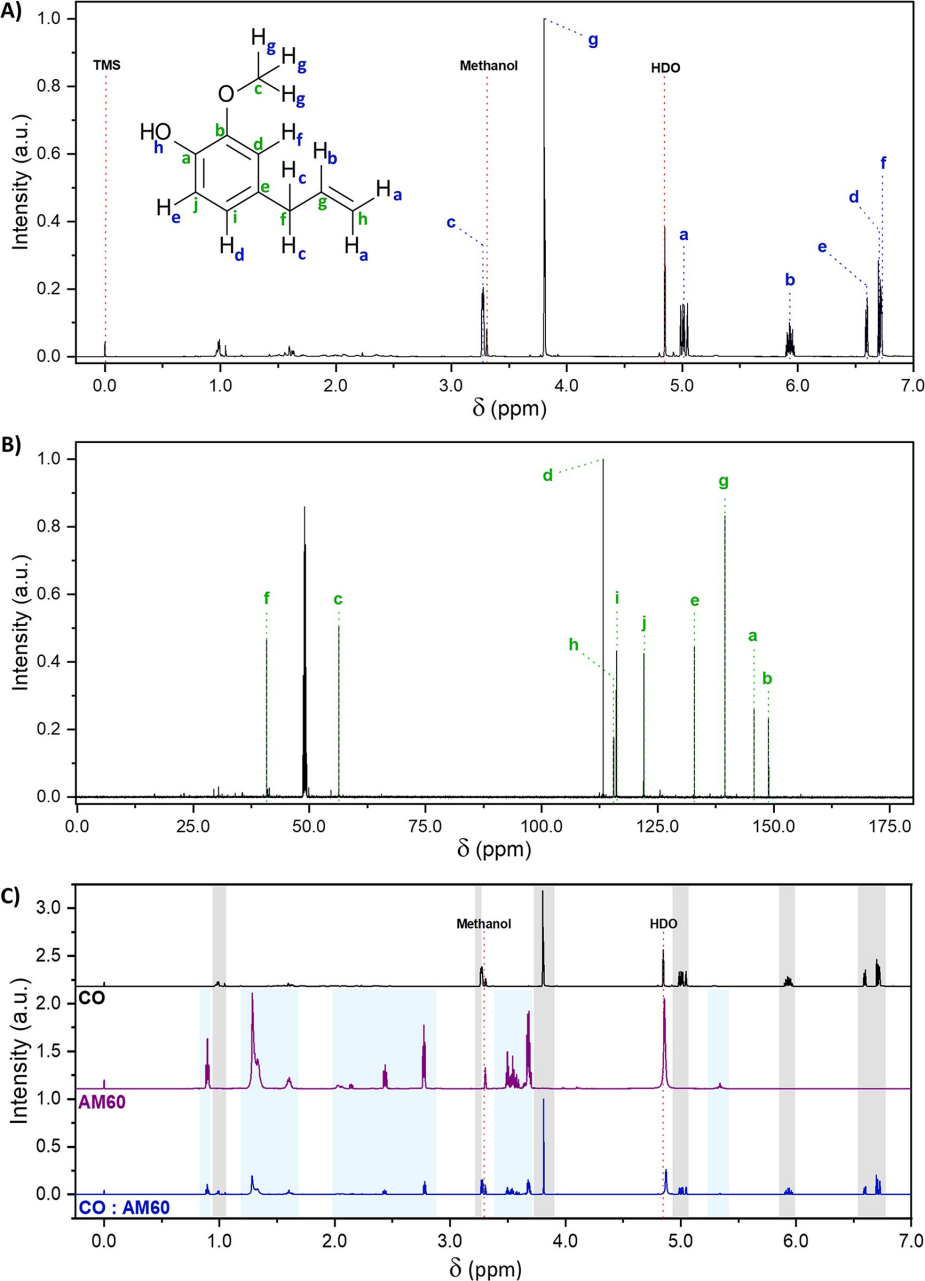
^1^H and ^13^C NMR spectra. **(A)** ^1^H NMR, **(B)** ^13^C NMR spectra of clove oil (CO) obtained at 600 MHz in CD_3_OD, and **(C)** ^1^H NMR spectra of clove oil (CO), amide 60 (AM60), and 1:1 CO:AM60 binary mixture.

**Fig 4:**
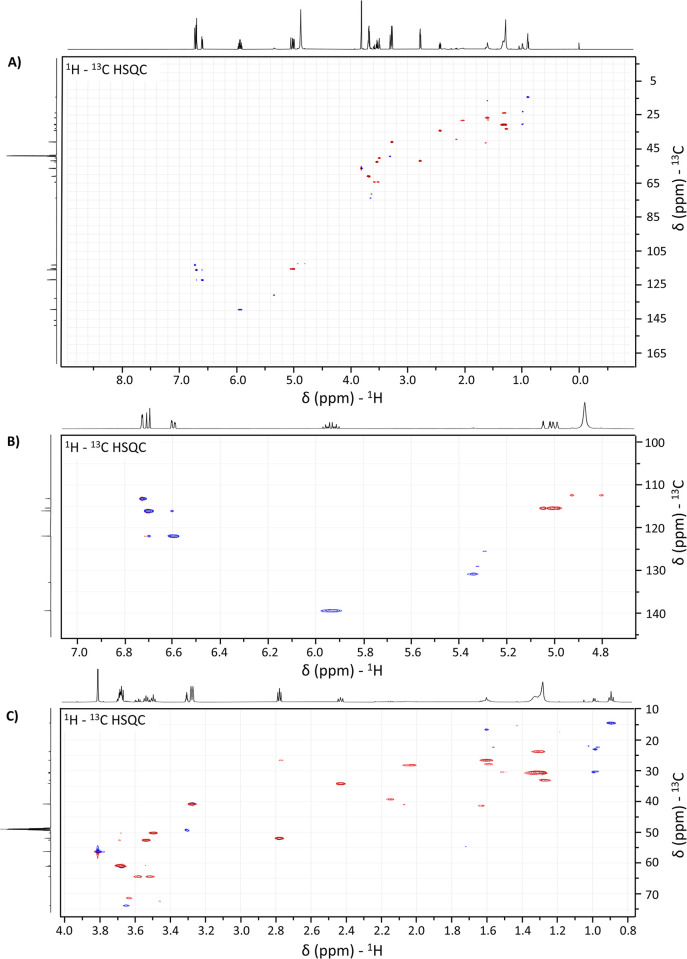
^1^H–^13^C Heteronuclear Single Quantum Correlation spectrum of CO:AM60 binary mixture. Full ^1^H – ^13^C HSQC spectra of the 1:1 binary mixture of CO and AM60 **(A)**. Zoom regions for correlations between **(B)** ^1^H signals from 4.7 to 7.0 ppm with ^13^C signals from 100 to 145 ppm and **(C)** ^1^H signals from 0.8 to 4.0 ppm with ^13^C signals from 10 to 75 ppm.

The ^1^H and ^13^C NMR spectra of CO are shown in Fig 3, with a detailed assignment of each observed signal. The most intense signals reveal the eugenol structure. The results of ^1^H–^13^C HSQC (Fig 4) and DEPT-135 (S2 Fig) were also considered to ensure a precise assignment of each signal. DEPT-135 allowed to verify that such more intense signals were not associated with the isoeugenol isomer but rather with eugenol, given the presence of the CH2 groups, evidenced by negative signals at 40.0 and 115.4 ppm.

The assignments detailed in Fig 3 agree with previously reported literature [49], differing only by inversed attribution of the "d" and "h" and "i" and "j" carbons, which could be confirmed by the aid of DEPT-135 and HSQC.

In the ^1^H-NMR spectrum of the AM60 (Fig 3C), the characteristic signals of the coconut fatty acid diethanolamide can be found, highlighted in the blue-shadowed regions and were assigned according to reported by Leng, et al. (2022) [50]. Namely, the signal at 0.89 ppm and those between 1.24–1.63 ppm are associated with the aliphatic portion of the fatty acid chain, while the signals comprised between 2.00 and 2.80 ppm are related to the CH_2_ hydrogens of the H_2_C–N(R)–CH_2_ from the diethanolamide portion and those between 3.45–3.65 ppm, related to the CH_2_ groups bonded to the same fragment (H_2_C–H_2_CN(R)CH_2_–CH_2_). Additionally, the signals at 3.70 and 5.34 were respectively assigned to the terminal hydroxyl groups and the hydrogens of HC = CH bonds in the region between the carbonyl of the amide and the fatty acid aliphatic chain.

To better investigate the possible changes in the structure of the eugenol, the ^1^H – ^13^C correlation spectrum was obtained (Fig 4). From the correlation spectrum, it was possible to observe 7 strong correlations between carbons and hydrogens for those signals previously assigned to the eugenol structure. This result was consistent with the expected for eugenol since the structure shows 3 carbons that are not bonded with any hydrogen.

In the Fig 4B, the following correlations were observed: i) 5.94 with 139.5 ppm; ii) 6.60 with 122.0 ppm; iii) 6.70 with 116.1 ppm; iv) 4.99–5.05 with 115.4; v) 6.72 with 113.2 ppm. Additionally, two correlations associated with the eugenol structure were observed in the zoom region of Fig 4C: the 3.81 with 56.0 ppm and 3.17 with 40.0 ppm. All these correlations are in accordance with the assignments presented in Fig 3. No correlations were observed for those 13C signals at 132.0, 145.8, and 148.8 ppm. Also, those signals were not observed in DEPT-135, confirming that they are related to the carbons with no hydrogens.

In conclusion, all the characteristic signals of the eugenol and AM60 were preserved after mixing CO and AM60. Therefore, the color change observed for CO:AM60 is not expected to result from a chemical reaction that could affect the eugenol structure. However, it can be suggested that the orange color observed derives from the formation of the strong acid-base pair interaction between eugenol and diethanolamine, that can strongly affect the electronic transitions. In this context, red-shifts in the phenol electronic transitions were already observed when it is mixed with mono, di and triethanolamine [51].

Based on that, all the materials were considered compatible with CO and were used in formulation development.

### 3.2. Microemulsion development

Surfactants commonly used in sanitizer products and approved by the preformulation studies (RX95 and AM60) were selected to formulate a CO-loaded microemulsion. Three initial ratios of CO:RX95:AM60:water were prepared to optimize the formulation, as outlined in the S2 Table. The selection criteria involved choosing the formulation with a smaller particle size and lower polydispersity. The same dilution was used for characterizing all the microemulsions, ensuring a count rate higher than 100 kcps [52,53]. Microemulsion 2 presented the lowest particle size and PDI, emerging as the most suitable for developing sanitizer tablets (Table 1).

**Table 1:** Average intensity of hydrodynamic diameter (Z-average), polydispersity index (PDI), and zeta potential (mV).

Sample	Z-average (nm)	PDI	Zeta potential (mV)
Microemulsion 1	230	0.330	-32.5
Microemulsion 2	100	0.316	-19.5
Microemulsion 3	245	0.358	-38.0

### 3.3. Acaricidal activity and larvae mortality

The natural product CO showed potent acaricidal and larvicidal effects corroborating the literature [6,7], even superior to the commercial product Triatox® used as a positive control (Table 2). In fact, the treatment with this essential oil demonstrated the highest efficacy, with 95.0% mortality of females and an impressive 99.9% product efficacy. Additionally, this sample exhibited the lowest reproductive efficiency (61) and a larvae mortality of 100%.

**Table 2:** Acaricidal activity and larvae mortality of in vitro treatment against *R*. *microplus* from naturally infested cattle.

Treatment	Female mortality (%)	Hatching (%)	Reproductive efficiency	Product efficacy (%)	Larvae mortality (%)
Water	10.0	100.0	117095	-	-
Triatox^®^	13.3	100.0	99781	35.7	53.0
CO	95.0	0.0	61	99.9	100.0
RX95:AM60 (3:1)	6.6	100.0	98490	15.9	-
CO microemulsion	70.0	0.0	85	99.9	94.0

Furthermore, the microemulsion containing such essential oil exhibited a similar product efficacy, a high female mortality rate of 70%, and a larvae mortality rate of 94%. The surfactants used in the test (RX95:AM60 3:1), in turn, presented results similar to the water, evidencing that they did not play a significant role in the acaricidal and larvicidal activity, which can be entirely attributed to the CO. According to the results, the microemulsion did not interfere with the CO effect, making it a viable and highly effective alternative to controlling *R*. *microplus*, both in ticks and larvae.

### 3.4. Soap synthesis

The liquid soap was efficiently obtained through the saponification reaction of the recycled soy oil with KOH, as described in the methodology section. The methodologies explored to produce the powder soap by spray dryer are outlined in (S1 Table). The primary challenge was identifying drying parameters conducive to creating a fine and flowable powder. Typically, such physical attributes are achieved through high-temperature drying processes, from 130 to 150°C [54,55], particularly when an aqueous vehicle is involved.

However, through drying methods 1 and 2 (S1 Table), which were carried out at 90°C inlet and 65°C outlet temperatures, no powder was obtained. Instead, a soap film was formed in the cyclone unit. Such an observation can be understood based on the previously discussed DSC results, which have evidenced three endothermic events at regions of 40°C, 95°C, and 115°C, probably associated with the melting of some fatty acids and residual triglycerides from the saponification reaction. Hence, based on these results, it was determined that the soap could not be exposed to high temperatures in the drying process.

At lower temperatures, the complete removal of water from the system was unattainable. The residual water post-atomization, combined with a possible amount of glycerin (a natural by-product of the saponification reaction), acted as a plasticizer, forming a soap film inside the equipment by coating its surface.

Subsequent attempts added excipients to enhance the drying process. Two liquids diluents (water and ethanol), and a solid silica (Aerosil®) were selected for this purpose. The concentration of these materials was tailored based on the solids content present in the raw soap, which was 16.49% ± 0.01. Consequently, various proportions of liquid and solid additives were employed, together with adjustments in drying parameters (S1 Table).

**Fig 5:**
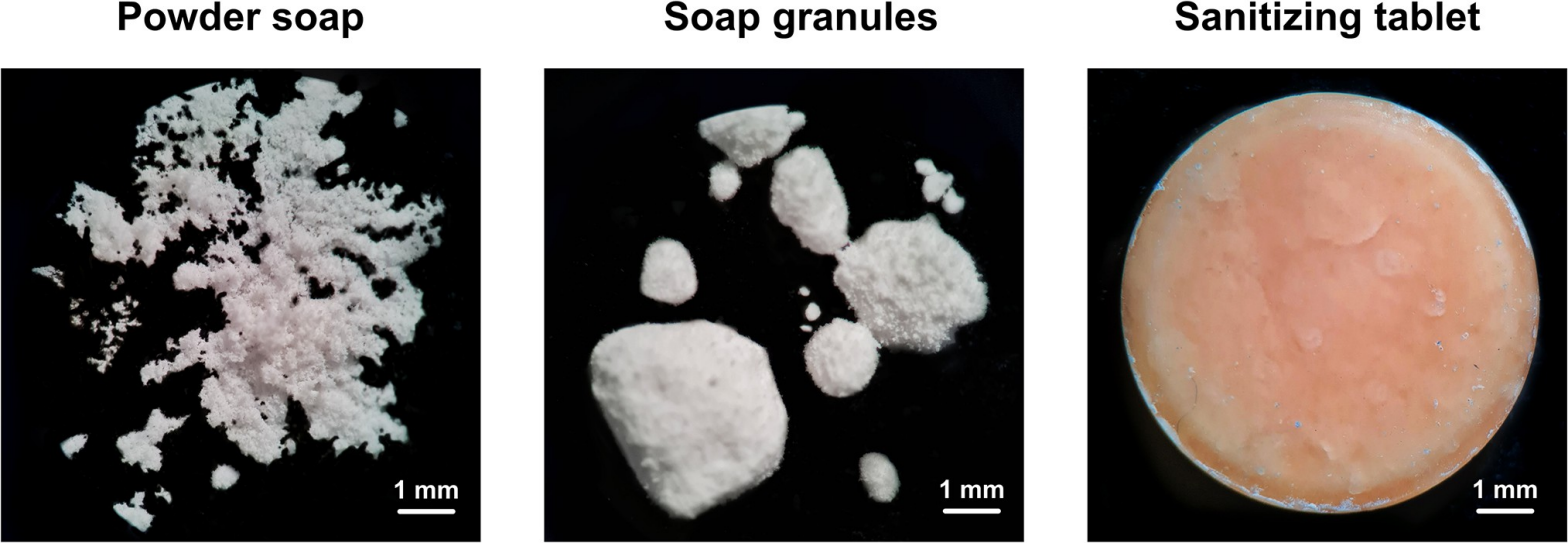
Optical microscopy images. Photographs obtained for the powder soap, granules, and sanitizer tablet at a 4.5 × magnification.

Finally, methods 1 to 8 presented the described film-forming issue, particularly in the cyclone unit, rendering the recovery of any powder unfeasible. In contrast, Method 9 successfully generated a substantial amount of powder with attributes conducive to compression. Indeed, the incorporation of high concentrations of ethanol and Aerosil® facilitated the complete drying at a temperature of 50°C. The low injection flow also ensured a continuous powder formation through the droplet drying process, preventing film formation within the equipment. The resulting powder is shown in Fig 5.

### 3.5. Sanitizer tablet development

The powder was blended with the CO microemulsion to create a non-cohesive granule with adequate compatibility, ensuring effective powder compaction. Indeed, in powder compression, enhancing the material flow and compressibility properties is often necessary to achieve uniform die-filling and form a sturdy solid structure in a tablet press. Such characteristics can be accomplished by transforming fine powders into larger agglomerates, i.e., granules [56,57].

In this scenario, a wet granulation process was implemented by introducing the microemulsion to the powder. In this process, the microemulsion acts as a binder, bringing together small soap powder particles and creating larger granules. Given the high volatility of the main component (eugenol), the drying stage of the wet granulation process was suppressed. Additionally, a small amount of microemulsion was added to facilitate powder agglomeration without excessive wetness. The soap:microemulsion ratio of 1:1 (w/v) met these criteria and was employed for tablet production. Moreover, the resulting granules exhibited varied sizes and a spherical morphology (Fig 5), ideal for the compression process ensuring the filling of intergranular voids [56].

The absence of drying in the granulation process and oily material in the granules led to particle sintering during compression, resulting in a slightly porous surface (Fig 5). This outcome proved advantageous, facilitating the wetting of these tablets. In fact, the material promptly dispersed in water at the specified application proportion (0.25 g in 250 mL of water; 1:750; w/v). S3 Fig illustrates the soap granule, the tablet, and its dispersion in water, forming a colloidal suspension.

This system was subjected to analysis through DLS and electrophoretic mobility. Additionally, the dispersion of the microemulsion and powder soap, individually, was analyzed at the same dilution as the final product (1:750; w/v) in order to determine whether the colloidal dispersion of the tablet resulted from dispersing the microemulsion, the soap, or a combination of both.

**Fig 6:**
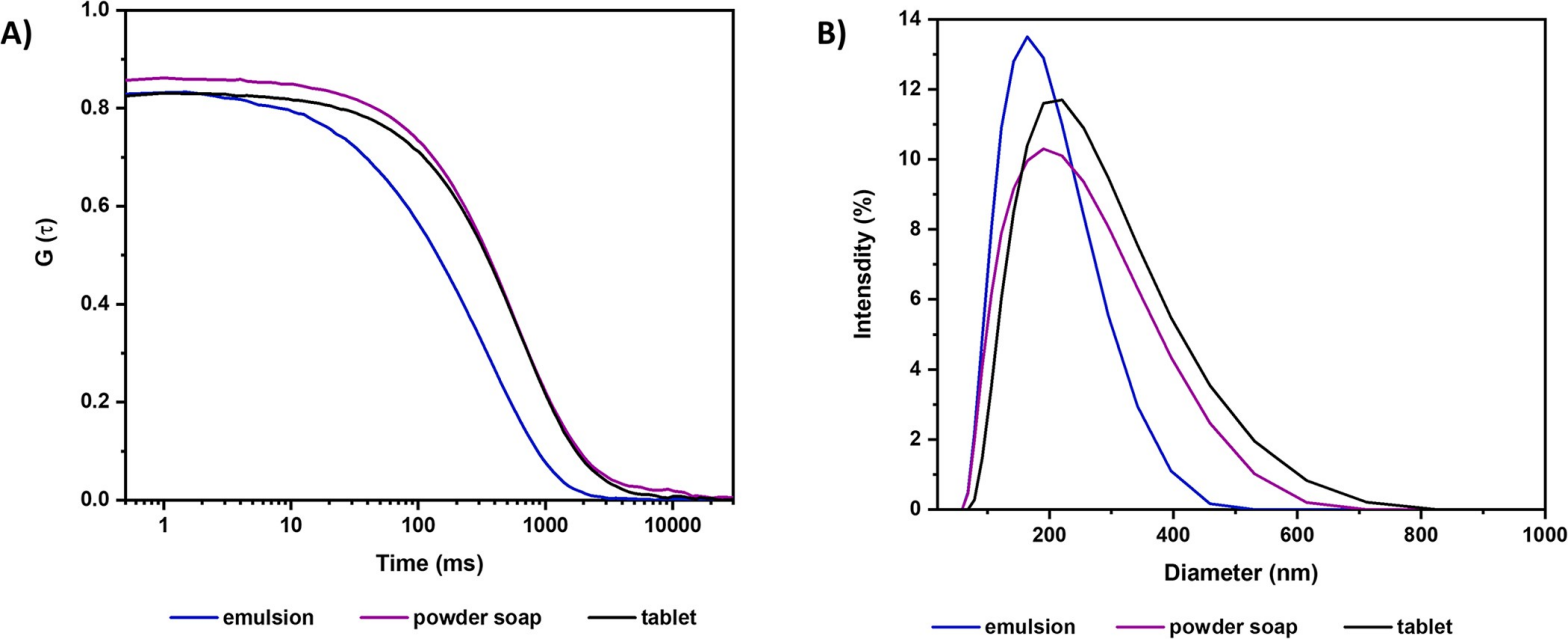
Results of Dynamic Light Scattering (DLS) analyses. **(A)** Correlation function and **(B)** hydrodynamic diameter distribution of the micro-emulsion, powder soap, and sanitizer tablet dispersed in water at the final product dilution (1:750 w/v).

In Fig 6A, the correlation curve of the samples is depicted, with maximum amplitude values of 0.82, 0.85, and 0.83 for the microemulsion, soap, and tablet, respectively. Considering the theoretical limit is 1.0, these results indicate a high resolution in the analysis. All samples exhibit a broad peak, indicating significant polydispersity among the formed nanostructures (Fig 6B).

The mean intensity value (Z-average) represents the intensity-weighted mean hydrodynamic size of the ensemble collection of identified nanostructures derived from the correlation curve shown in Fig 6A. The primary population of droplets or particles in the microemulsion, soap, and tablet samples has sizes of 100, 214, and 221 nm, respectively (Table 3).

**Table 3:** Average intensity of hydrodynamic diameter (Z—average), polydispersity index (PDI), and zeta potential (mV) of the chosen emulsion formulation, the powder soap, and the sanitizer tablet diluted in water using the same dilution as the final product (tablet).

Sample	Z-average (nm)	PDI	Zeta potential (mV)
CO microemulsion	100	0.316	-19.5
Powder soap	214	0.338	-1.5
Sanitizer tablet	221	0.510	-17.2

The zeta potential value for the microemulsion was -19.5 mV, which indicates that this product could have short-term stability since values above 30 mV in modulus, even in the range of 60 mV, are desirable for stable products [58]. In this scenario, incorporating such microemulsion in a compacted solid matrix seems more adequate to preserve the stability of the formulation, avoiding phase separation and the evaporation of the essential oil.

Notably, the size of the nanoparticles formed in tablet suspension is primarily influenced by the soap, as evidenced by the similar Z-average values, 221 and 214 nm, for the tablet and powder soap, respectively (Table 3). Conversely, the zeta potential of the sanitizer tablet is predominantly determined by the microemulsion, which exhibited values of -17.2 and -19.5 mV, respectively (Table 3).

Given the non-ionic nature of the surfactants and other materials employed, the obtained high negative zeta potential result was unexpected. The observed electrostatic attraction in the slipping plane of the microemulsion corroborates the proposed deprotonation of eugenol outlined in the preformulation studies. Such occurrence is facilitated by the presence of AM60, leading to an augmented concentration of negative charges on the surface of the microemulsion droplets, resulting in a more negative zeta potential.

This phenomenon, observed in the preformulation study and confirmed by the microemulsion’s change in color, contributes to the droplets’ electrostatic stability, preventing their aggregation and phase separation. The persistence of this behavior in the tablet is advantageous for its nanostructure stability in the colloidal state.

The average weight of the tablets was 250.05 ± 0.38 mg, with a coefficient of variation of only 0.2%. Additionally, the friability was 0.6%, representing a high level of mechanical resistance in the sanitizer tablets. This attribute supports its commercial viability and contributes to its overall stability. Indeed, the low friability value is likely attributed to the particle sintering facilitated by the microemulsion in the formulation. Furthermore, the robust mechanical resistance was confirmed by the hardness test, yielding a value of 6.4 ± 0.2 kgF. The associated coefficient of variation of 3.5% highlights the reproducibility of the sintering mechanism, ensuring consistent mechanical strength.

Finally, the eugenol content was measured in the sanitizer tablet. The acid-base interaction of the phenolic group in eugenol with diethanolamine, previously observed in the preformulation studies, was explored for determining eugenol concentration using UV-Vis spectrophotometry. This approach was chosen since the eugenol band in the isolated CO is detectable at 280 nm [59], a wavelength at which other excipients also exhibit absorption. However, in the presence of AM60, CO absorbs light in the visible region at maximum wavelength of 493 nm (S4A Fig), where no excipient shows other absorptions. Thus, it was possible to selectively detect eugenol and quantify its concentration in the tablet.

In this approach, only eugenol displayed a signal at 493 nm. Accordingly, this wavelength was used to construct the calibration curve from eugenol and AM60 (S4 Fig). The eugenol content within the sanitizer tablet was subsequently determined to exhibit a concentration of 95.5% ± 3.3 (S4A Fig).

### 3.6. Determination of antimicrobial activity

Eugenol is the main component of CO. The low MIC, MBC, and MFC values confirmed the potent antimicrobial activity of isolated eugenol, i.e., 0.009 mg mL^-1^ for *S*. *aureus* and 0.006 mg mL^-1^ for *E*. *coli* and *C*. *krusei* (Table 4). Previous studies reported high activity of this compound against several bacteria and fungi. In bacteria, the eugenol antimicrobial action is due to the alteration of membrane permeability, followed by ion leakage and loss of other cellular contents, resulting in cell death. Regarding the mechanism of action against fungi, eugenol induced in *C*. *albicans* an oxidative stress response characterized by increased levels of superoxide dismutase and consequently elevated production of intracellular reactive oxygen species, which caused lipid peroxidation of the cytoplasmic membranes and cell ***death*** [60].

**Table 4:** Minimum inhibitory concentration (MIC), *minimum* bactericidal concentration (MBC), and minimum *fungicidal* concentration (MFC) of eugenol standard, CO, microemulsion, and sanitizer tablet.

Sample	*S*. *aureus* ATCC 25923		*E*. *coli* ATCC 25922		*C*. *krusei* ATCC 6258	
	MIC (mg mL^-1^)	MBC (mg mL^-1^)	MIC (mg mL^-1^)	MBC (mg mL^-1^)	MIC (mg mL^-1^)	MFC (mg mL^-1^)
Eugenol	0.009	0.009	0.006	0.006	0.006	0.006
CO	0.250	0.500	0.250	0.250	0.063	0.125
CO microemulsion	0.070	0.070	0.050	0.050	0.050	0.050
Sanitizer tablet	4.170	6.250	5.000	8.330	4.170	6.250

CO, in turn, presented a much less potent antimicrobial activity compared to eugenol. The attenuated antimicrobial effect in the essential oil may be related not only to the dilution of eugenol [61], but also to the low availability of this active ingredient in the pure essential oil. However, the MIC and MBC values of CO from the present study against S. aureus (0.250 and 0.500 mg mL^-1^) and *E*. *coli* (0.250 mg mL^-1^) were lower than the results reported by Bai et al. (2023) [61], who obtained MIC and MBC values of CO against *S*. *aureus* of 0.520 and 1.040 mg mL^-1^ and *E*. *coli* of 0.640 and 1.280 mg mL^-1^. The MIC and MFC values of CO from this study against *C*. *krusei* (0.063 and 0.125 mg mL^-1^) were also lower than the results reported by Biernasiuk et al. (2023) [62], who obtained MIC and MFC values of CO against *C*. *krusei* of 0.500–2.000 and 1.000–4.000 mg mL^-1^, respectively.

It was observed an increase in the antimicrobial activity of CO microemulsion with MIQ, MBC, and MFC values of 0.050–0.070 mg mL^-1^ compared to pure CO (MIQ of 0.063–0.250 mg mL^-1^ and MBC of 0.125–0.500 mg mL^-1^) against *S*. *aureus*, *E*. *coli* and *C*. *krusei* (Table 4). Thus, the emulsification of CO considerably improved its antimicrobial action. This improvement is likely attributed to surfactants in the microemulsion, which facilitate the cellular permeability of eugenol, thereby amplifying its antimicrobial efficacy (Bai et al., 2023). Notably, the emulsification of CO has been previously recognized as a technological approach to enhance natural compounds’ antimicrobial activity [63].

Importantly, in the microemulsion, the CO content is 12.5%. Consequently, when converting the MIC, MBC, and MFC values of the microemulsion to equivalent CO values, the results are corrected from 0.070 mg mL^-1^ to 0.009 mg mL^-1^ (MIC and MBC for *S*. *aureus*) and from 0.050 mg mL^-1^ to 0.006 mg mL^-1^ (MIC, MBC, and MFC for *E*. *coli* and *C*. *krusei*). Therefore, emulsifying the essential oil enables the CO to exert the same antimicrobial potency as the isolated eugenol.

The MIC, MBC, and MFC values were notably higher for the sanitizer tablet than the other samples analyzed. However, relativizing the results according to the concentration of CO in the tablets, which is 6.25%, the values for the antimicrobial activity test are corrected to MIC value of 0.260 mg mL^-1^ for *S*. *aureus*, 0.312 mg mL^-1^ for *E*. *coli*, and 0.260 mg mL^-1^ for *C*. *krusei*. Similarly, the corrected MBC is 0.390 mg mL^-1^ for *S*. *aureus* and 0.520 mg mL^-1^ for *E*. *coli*, while the corrected MFC is 0.390 mg mL^-1^ for *C*. *krusei*.

Given these results, it is concluded that the emulsification of CO renders the antimicrobial action of the formulation very similar to that of the isolated eugenol compound. At the same time, incorporating the emulsion in a soap matrix aligns the antimicrobial action of the sanitizer closely with that of the essential oil. Considering the more promising antibacterial action of the sanitizer tablet, a disk diffusion test was assessed using the bacteria species *S*. *aureus* and *E*. *coli*.

**Fig 7:**
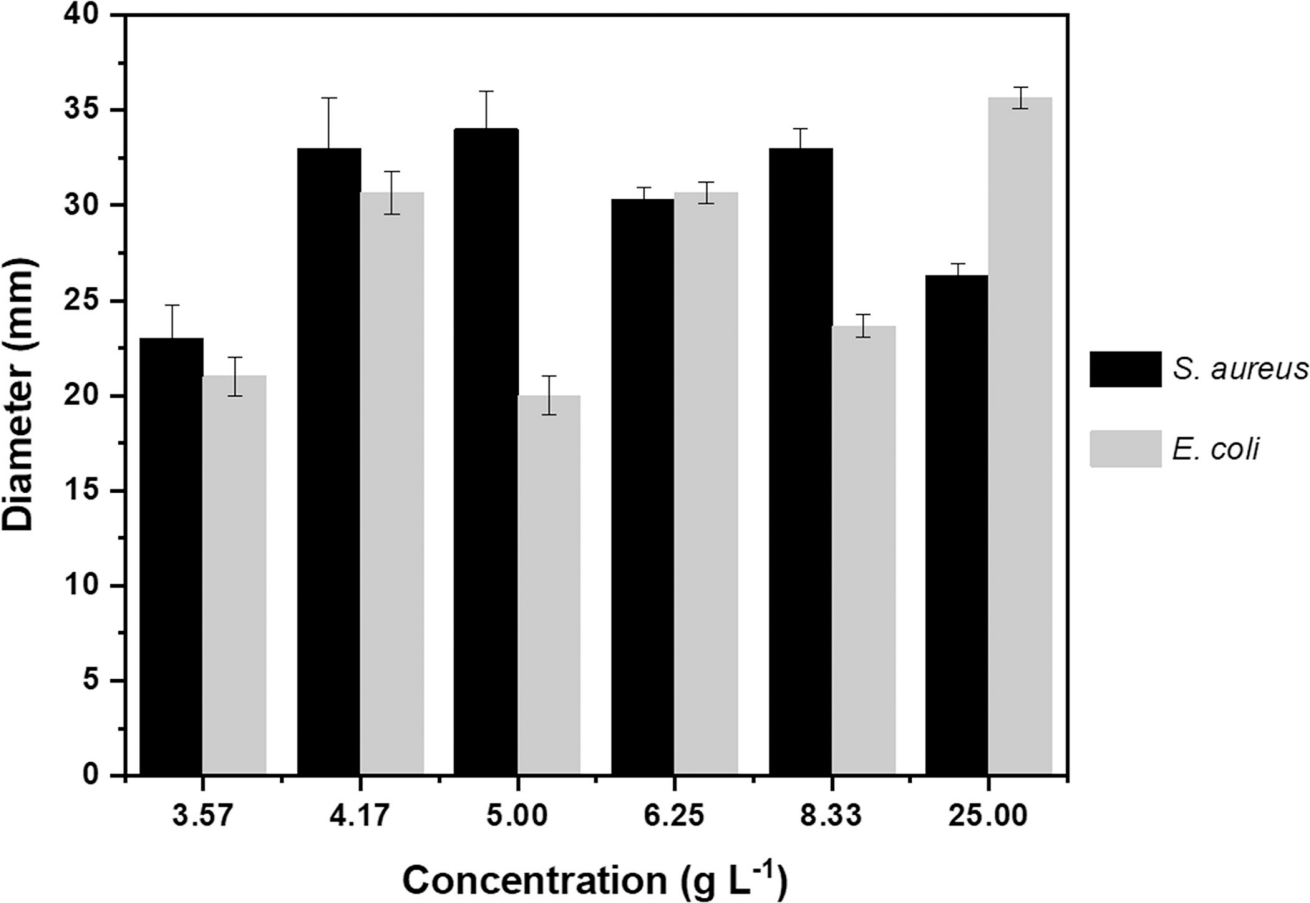
Antibacterial efficacy of the sanitizer tablet. Disk diffusion method results showing the antibacterial activity of the tablet against *S*. *aureus* and *E*. *coli*.

Fig 7 illustrates the antimicrobial activity achieved through the disk diffusion method for the sanitizer tablet at various concentrations. Microbial growth inhibition halos were obtained in all the different dilutions tested of the sanitizer tablet (3.57, 4.17, 5.00, 6.25, 8.33, and 25.00 g L^-1^), with values of 23.5 to 34.0 mm for *S*. *aureus* and 20.0 to 34.5 mm for *E*. *coli*, showing that even in the highest dilutions the sanitizer tablet showed appreciable antimicrobial activity. The results of this study with CO-microemulsion are comparable to studies that used CO. Kacániová et al. (2021) obtained an inhibition zone of 15.8 mm for *S*. *aureus* using pure CO and Ginting et al. (2021) obtained an inhibition zone of 24.3 mm for *E*. *coli* using CO diluted to 40% [64,65].

The combination of these features makes the developed product completely innovative and versatile. In fact, the high eugenol content in CO (above 80%) allows for a reduced quantity of oil in formulations, significantly lowering production costs while maintaining high efficacy. Additionally, CO’s potent antimicrobial, acaricidal, and larvicidal properties are well documented both in this study and in previous research [66–68], making this active component especially attractive, compared to many other essential oils. Finally, most commercially essential oil-based sanitizers are formulated as aqueous solutions or emulsions, which face challenges due to the volatile nature of these oils, leading to rapid evaporation [66]. The formulation presented here innovates by incorporating CO into a stable microemulsion-based tablet, a solid matrix that overcame these limitations, providing a novel and more efficient method for delivering antimicrobial and pest control benefits in a convenient solid form.

## 4. Conclusions

Developing an effective and stable product containing essential oils is challenging, especially when different manufacturing processes are involved. In this context, the development of CO-based sanitizer tablets started with preformulation studies using thermal and NMR analyses that confirmed the compatibility of this natural product with the selected formulation materials.

Obtaining a CO microemulsion, in turn, allowed its stabilization, in addition to maintaining the promising acaricidal and larvicidal activity of this natural product. Then, a detailed process of optimization of the soap by spray-drier was carried out in order to obtain an adequate matrix to incorporate the CO microemulsion by a wet granulation process without heating. The granules preserved the characteristics of the microemulsified system, including its colloidal properties. Finally, the sanitizer tablets produced from such granules resulted in a uniform product with adequate physical-chemical properties and rapid dispersion. Antimicrobial activity tests demonstrated the effectiveness of the sanitizer tablet against bacteria and fungi, exhibiting comparable antimicrobial potency to isolated CO.

This sanitizing tablet technology has broad applications. In healthcare, pharmaceuticals, and food industries, it can be used for effective surface disinfection. In agriculture and animal husbandry, its acaricidal and larvicidal properties could be explored in controlling pests that affect livestock and crops. Additionally, veterinary care and households can leverage its convenience and stability for everyday cleaning and pest control. In summary, the sanitizer tablet developed with cutting-edge technology is a natural product with rapid water dispersion and practical application, representing a potential alternative for effective antimicrobial and pest control.

## Supporting information

S1 Fig Clove oil and amide 60 mixture. The 1:1 mixture of CO and AM60 exhibiting a notable change in color, transforming the originally practically colorless components into an orange mixture.
(DOCX)

S2 Fig NMR spectra of 1:1 binary mixture of CO and AM60. (a) 1H NMR, (b) 13C NMR and (c) DEPT-135, all spectra were obtained at 600 MHz in CD_3_OD.
(DOCX)

S3 Fig From granulation to water dispersion. Representation of the granulate formed by powder soap and clove oil emulsion, the developed sanitizer tablet and its dispersion in water.
(DOCX)

S4 Fig UV-Vis spectra analysis of tablet components and sanitizer tablet highlighting new eugenol band at 493 nm. (a) UV-vis spectra of the tablet components without AM60 and CO (powder soap, RX95, and AM60), CO with AM60, highlighting the new eugenol band that appears at 493 nm, and of the sanitizer tablet. (b) UV-Vis spectra for CO and AM60 showing the new band (493 nm) at different concentrations and (c) the respective adjusted curves at λmax.
(DOCX)

S1 Table Drying methods for saponified liquid matrix tested in a spray dryer to obtain powder soap. The table lists the tested methodologies, the proportion of soap and the liquid or solid additives used, along with the drying parameters employed in the equipment.
(DOCX)

S2 Table Emulsion compositions. Initial concentration (%) of each material (CO, RX95, AM60, water) selected for evaluation to determine the final formulation.
(DOCX)
