# Supplementary material for: Antimicrobial and acaricide sanitizer tablets produced by wet granulation of spray-dried soap and clove oil-loaded microemulsion
Source: PLoS One. 2024 Nov 11;19(11):e0313517. doi: 10.1371/journal.pone.0313517 (PMC11554217; doi:10.1371/journal.pone.0313517)
Supplement: S1 Fig — The 1:1 mixture of CO and AM60 exhibiting a notable change in color, transforming the originally practically colorless components into an orange mixture. (DOCX) [file pone.0313517.s001.docx]

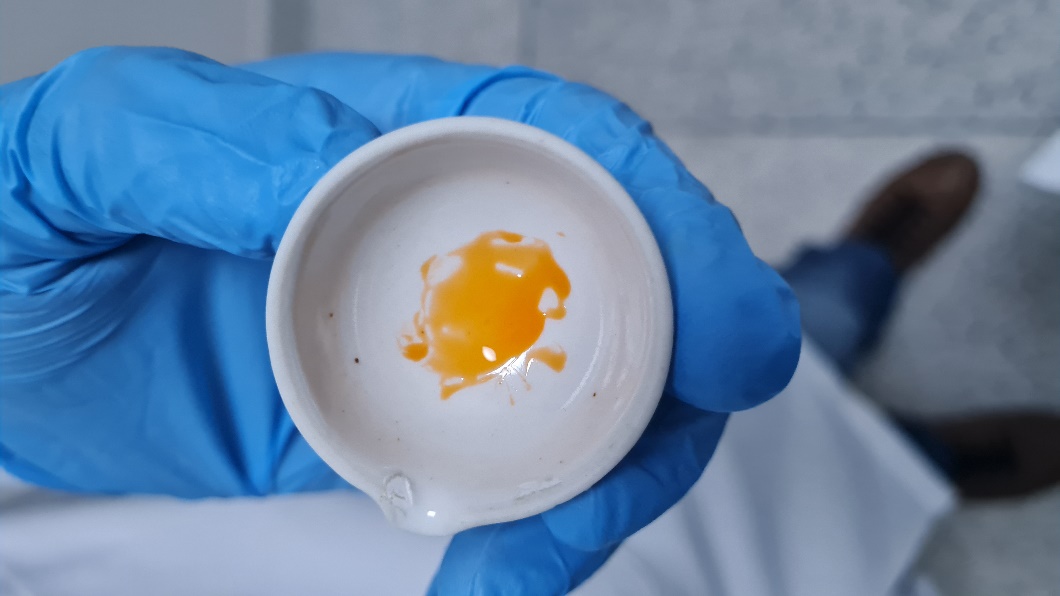


**S1 Fig. Clove oil and amide 60 mixture.** The 1:1 mixture of CO and AM60 exhibiting a notable change in color, transforming the originally practically colorless components into an orange mixture.
