# Supplementary material for: Antimicrobial and acaricide sanitizer tablets produced by wet granulation of spray-dried soap and clove oil-loaded microemulsion
Source: PLoS One. 2024 Nov 11;19(11):e0313517. doi: 10.1371/journal.pone.0313517 (PMC11554217; doi:10.1371/journal.pone.0313517)
Supplement: S2 Fig — (a) 1H NMR, (b) 13C NMR and (c) DEPT-135, all spectra were obtained at 600 MHz in CD3OD. (DOCX) [file pone.0313517.s002.docx]

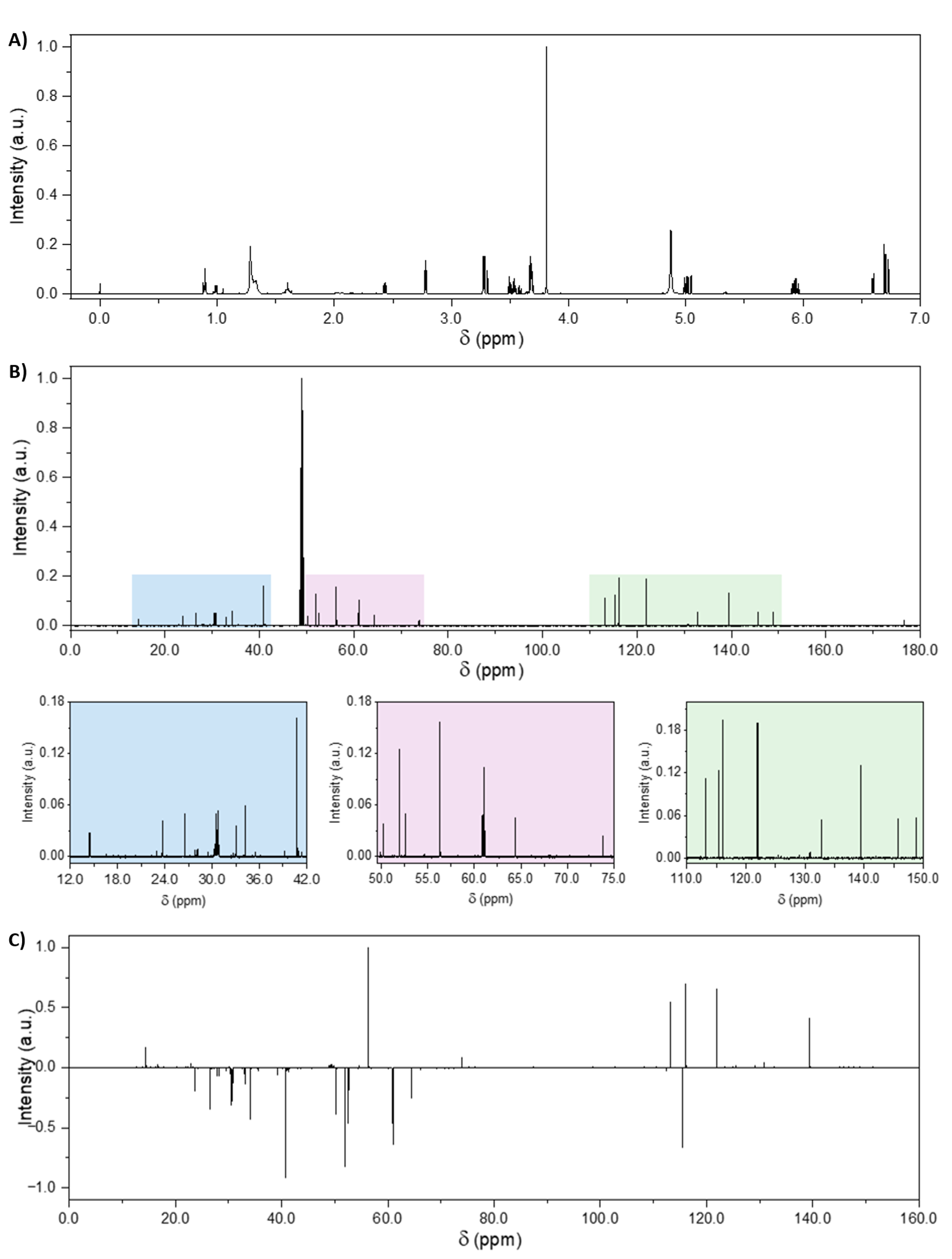


**S2 Fig. NMR spectra of 1:1 binary mixture of CO and AM60.** (a) 1H NMR, (b) 13C NMR and (c) DEPT-135, all spectra were obtained at 600 MHz in CD_3_OD.
