# Supplementary material for: Antimicrobial and acaricide sanitizer tablets produced by wet granulation of spray-dried soap and clove oil-loaded microemulsion
Source: PLoS One. 2024 Nov 11;19(11):e0313517. doi: 10.1371/journal.pone.0313517 (PMC11554217; doi:10.1371/journal.pone.0313517)
Supplement: S3 Fig — Representation of the granulate formed by powder soap and clove oil emulsion, the developed sanitizer tablet and its dispersion in water. (DOCX) [file pone.0313517.s003.docx]

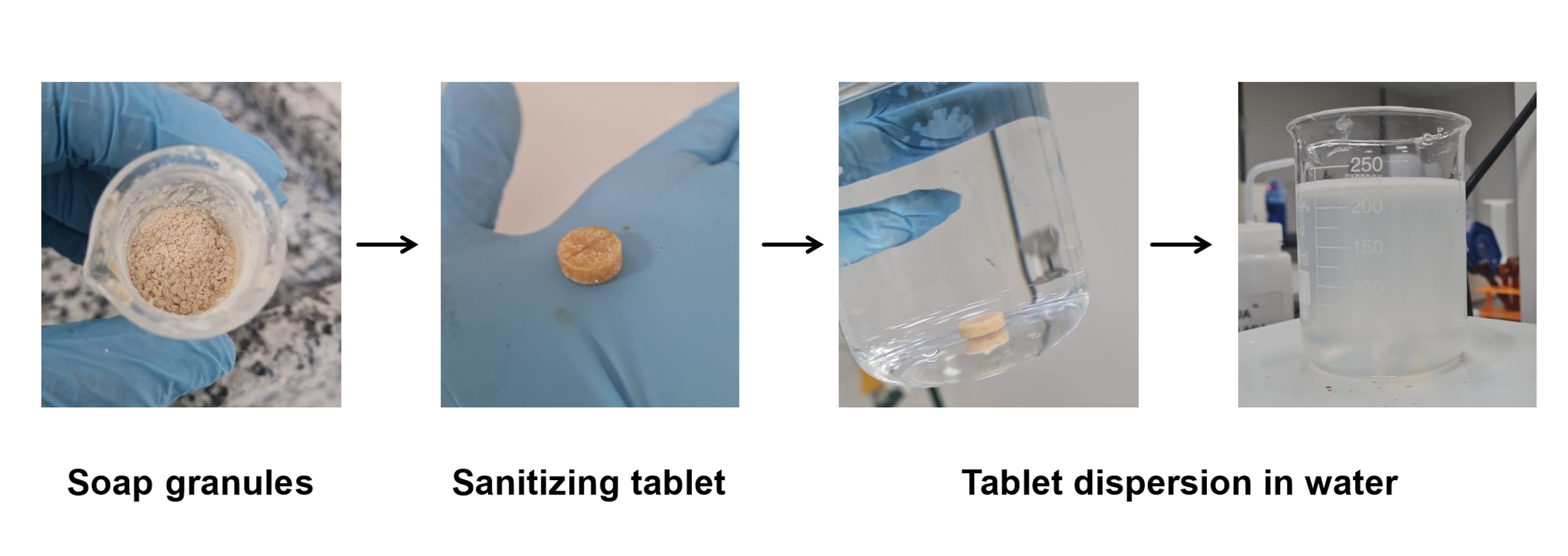


**S3 Fig. From granulation to water dispersion.** Representation of the granulate formed by powder soap and clove oil emulsion, the developed sanitizer tablet and its dispersion in water.
