# Supplementary material for: Antimicrobial and acaricide sanitizer tablets produced by wet granulation of spray-dried soap and clove oil-loaded microemulsion
Source: PLoS One. 2024 Nov 11;19(11):e0313517. doi: 10.1371/journal.pone.0313517 (PMC11554217; doi:10.1371/journal.pone.0313517)
Supplement: S4 Fig — (a) UV-vis spectra of the tablet components without AM60 and CO (powder soap, RX95, and AM60), CO with AM60, highlighting the new eugenol band that appears at 493 nm, and of the sanitizer tablet. (b) UV-Vis spectra for CO and AM60 showing the new band (493 nm) at different concentrations and (c) the respective adjusted curves at λmax. (DOCX) [file pone.0313517.s004.docx]

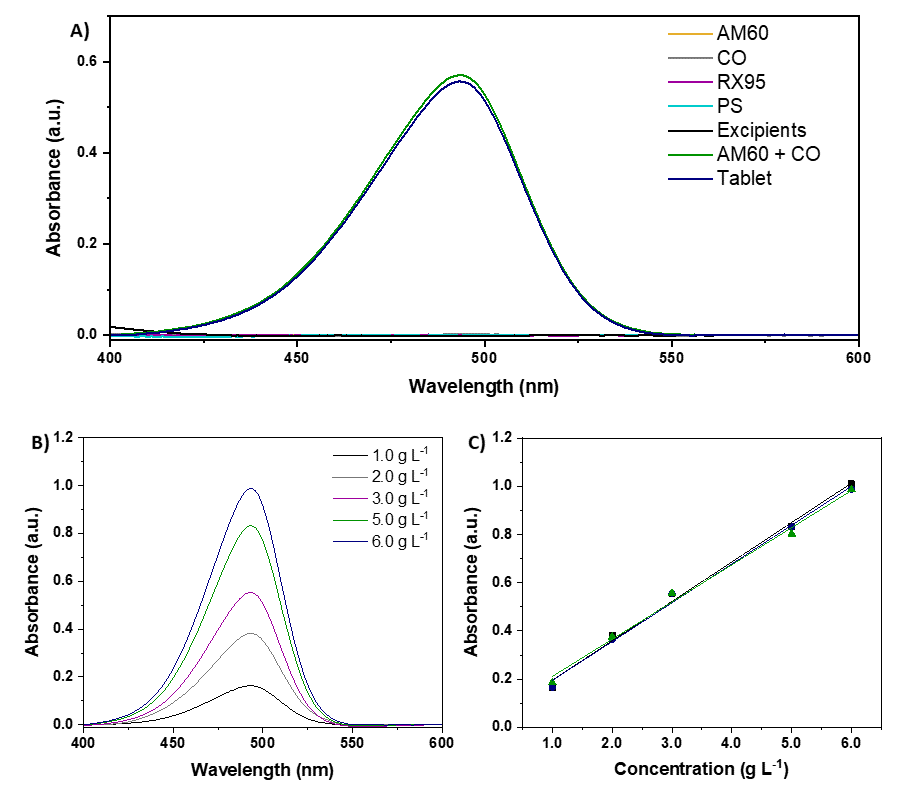


**S4 Fig.** **UV-Vis spectra analysis of tablet components and sanitizer tablet highlighting new eugenol band at 493 nm.** (a) UV-vis spectra of the tablet components without AM60 and CO (powder soap, RX95, and AM60), CO with AM60, highlighting the new eugenol band that appears at 493 nm, and of the sanitizer tablet. (b) UV-Vis spectra for CO and AM60 showing the new band (493 nm) at different concentrations and (c) the respective adjusted curves at λmax.
