# Supplementary material for: Antimicrobial and acaricide sanitizer tablets produced by wet granulation of spray-dried soap and clove oil-loaded microemulsion
Source: PLoS One. 2024 Nov 11;19(11):e0313517. doi: 10.1371/journal.pone.0313517 (PMC11554217; doi:10.1371/journal.pone.0313517)
Supplement: S1 Table — The table lists the tested methodologies, the proportion of soap and the liquid or solid additives used, along with the drying parameters employed in the equipment. (DOCX) [file pone.0313517.s005.docx]

**S1 Table.** **Drying methods for saponified liquid matrix tested in a spray dryer to obtain powder soap.** The table lists the tested methodologies, the proportion of soap and the liquid or solid additives used, along with the drying parameters employed in the equipment.

| **Method** | **Sample components** | | | | **Drying parameters** | | | | | | **Powder obtention** |
| --- | --- | --- | --- | --- | --- | --- | --- | --- | --- | --- | --- |
|  | **Liquid v/v (%)** | | | **Solid (w/v %)** | **Temperature (°C)** | | | **Aspiration (m^3^ min^-1^)** | **Injection flow (L h^-1^)** | **Air flow (mL min^-1^)** |  |
|  | ***Liquid Soap*** | ***Water*** | ***Ethanol*** | ***Silica*** | | ***Inlet*** | ***Outlet*** |  |  |  |  |
| 1 | 100 | - | - | - | | 90 | 65 | 1 | 0.4 | 50 | ✗ |
| 2 | 50 | 50 | - | - | | 90 | 65 | 1 | 0.4 | 50 | ✗ |
| 2 | 50 | 50 | - | - | | 50 | 40 | 1 | 0.4 | 50 | ✗ |
| 3 | 50 | 50 | - | 1 | | 50 | 40 | 1 | 0.4 | 50 | ✗ |
| 4 | 37.5 | 37.5 | 25 | 1 | | 50 | 40 | 1 | 0.4 | 50 | ✗ |
| 5 | 37.5 | 37.5 | 25 | 1 | | 50 | 40 | 1 | 0.4 | 50 | ✗ |
| 6 | 37.5 | 37.5 | 25 | 1 | | 60 | 40 | 1 | 0.4 | 50 | ✗ |
| 7 | 37.5 | 37.5 | 25 | 1 | | 50 | 40 | 1 | 0.25 | 50 | ✗ |
| 8 | 37.5 | 37.5 | 25 | 5 | | 50 | 40 | 1 | 0.25 | 50 | ✗ |
| 9 | 50 | 37.5 | 12.5 | 5 | | 50 | 40 | 1 | 0.25 | 50 | ✓ |
