# Supplementary material for: Antimicrobial and acaricide sanitizer tablets produced by wet granulation of spray-dried soap and clove oil-loaded microemulsion
Source: PLoS One. 2024 Nov 11;19(11):e0313517. doi: 10.1371/journal.pone.0313517 (PMC11554217; doi:10.1371/journal.pone.0313517)
Supplement: S2 Table — Initial concentration (%) of each material (CO, RX95, AM60, water) selected for evaluation to determine the final formulation. (DOCX) [file pone.0313517.s006.docx]

**S2 Table. Emulsion compositions.** Initial concentration (%) of each material (CO, RX95, AM60, water) selected for evaluation to determine the final formulation.

| **Sample** | **CO (%)** | **RX95:AM60 3:1 (%)** | **Water %** |
| --- | --- | --- | --- |
| Emulsion 1 | 5.0 | 45.0 | 50.0 |
| Emulsion 2 | 12.5 | 50.0 | 37.5 |
| Emulsion 3 | 20.0 | 46.7 | 33.3 |
